# Cannabis, Collaterals, and Coronary Occlusion

**DOI:** 10.1155/2011/469850

**Published:** 2011-07-06

**Authors:** Kalpa De Silva, Divaka Perera

**Affiliations:** Cardiovascular Division, Rayne Institute, St Thomas' Hospital, London SE1 7EH, UK

## Abstract

A 51-year-old gentleman, who regularly smoked cannabis, presented with chest pain and diaphoresis. He was haemodynamically stable. ECG showed ST depression, inferiorly, and 1 mm ST elevation in lead aVR. Emergent coronary angiography showed thrombotic occlusion of the left main coronary artery (LMCA), the dominant RCA provided Rentrop grade II collaterals to the LAD. The LMCA was successfully reopened by deployment of a bare-metal stent. Animal heart models suggest that endogenous cannibinoids may cause ischaemic preconditioning. This case suggests that the severity of ischaemia, and hence ECG changes and haemodynamic consequences following an acute occlusion of the LMCA, can be ameliorated by coronary collateralisation and possibly by preconditioning of the myocardium.

## 1. Case

**Figure 1:**
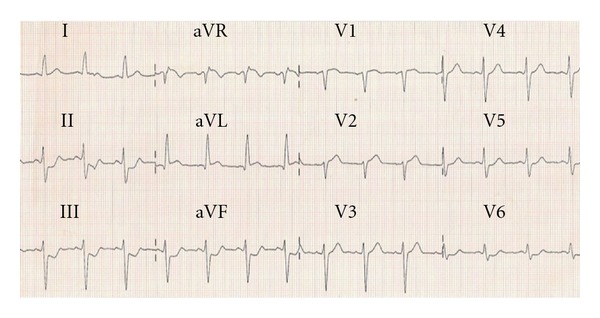
Presenting 12-lead electrocardiogram.

**Figure 2:**
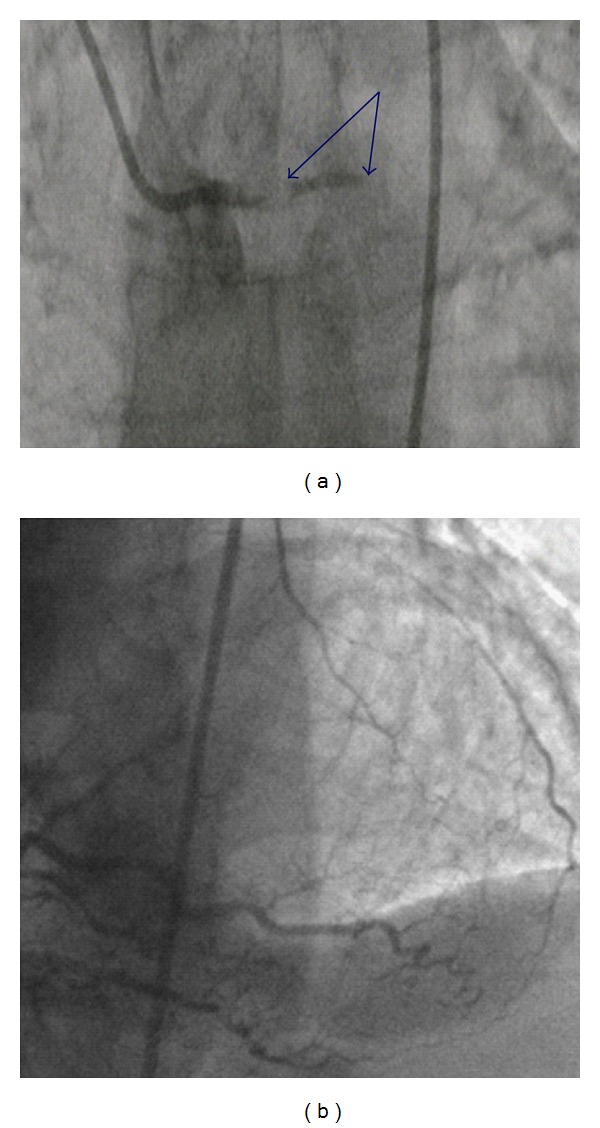
(a): Thrombotic occlusion of Left main coronary artery; (b): distal right coronary artery with collateral supply to left coronary system.

A 51-year-old gentleman presented to our hospital, with a one-hour history of chest pain and diaphoresis. He was a regular smoker of cannabis and tobacco but no other medical history of note. Despite being clammy, he had a regular pulse at 74 bpm, a blood pressure of 125/70 mmHg, and no evidence of cardiac decompensation. The electrocardiogram (ECG) showed 2 mm (0.2 mV) ST depression in leads II, III, aVF, and V6 and 1 mm ST elevation in leads aVR and V1 (Figure 1). Emergent coronary angiography showed complete thrombotic occlusion of the left main coronary artery (LMCA), with TIMI 0 flow (Figure 2). The dominant right coronary artery was unobstructed and provided Rentrop grade II collaterals to the left anterior descending artery (LAD).

**Figure 3:**
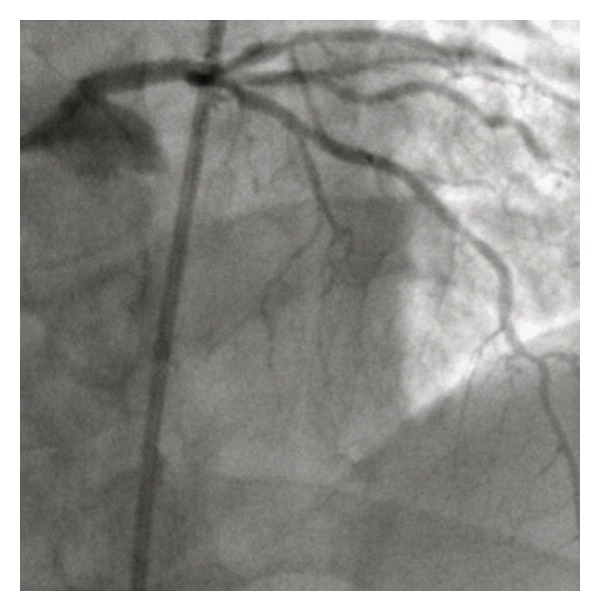
Left main coronary artery post-PCI.

We proceeded to treat the LMCA lesion by percutaneous coronary intervention with prophylactic intra-aortic balloon pump (IABP) support and a bolus of Abciximab. Restoration of flow by balloon angioplasty led to resistant ventricular fibrillation, which was successfully treated by repeated defibrillation and intravenous Amiodarone. A 3.0 × 18 mm bare-metal stent was deployed from the LMCA to the LAD and post-dilated with a 4 mm noncompliant balloon, achieving TIMI III flow in the LAD and circumflex arteries (Figure 3). The patient remained on IABP support for 24 hours, prior to being discharged after 5-day hospital admission. He remains well 6 months following this event, and echocardiography shows an ejection fraction of 40% with anterior hypokinesis. 

## 2. Discussion

The twelve-lead electrocardiogram (ECG) is a pivotal investigation for patients presenting with chest pain. The pattern of ischaemia on the ECG determines the mode and rapidity of revascularization and often indicates the infarct-related artery (IRA).

Delineating the presence of acute LMCA lesions can be difficult. ECG features of LMCA occlusion are said to include ST elevation in the anterior precordial leads, along with inferior ST segment shift and ST elevation in limb lead aVR. [1] The finding of lead aVR ST segment elevation being greater than or equal to lead V1 ST segment elevation is said to distinguish the LMCA group from the LAD group, with 81% sensitivity, 80% specificity, and 81% accuracy [2]. Previous reports by Engelen et al. [3] suggested that acute occlusion of the proximal left anterior descending artery (LAD), proximal to the first major septal branch, most frequently presented with ST segment elevation in lead aVR, due to the transmural nature of ischaemia to the basal septum. Further characterisation has demonstrated the presence of inferior ST segment depression during acute LMCA occlusions, independent of the presence of RCA stenoses, as a result of the profound ischaemia caused to the posterior aspect of the left ventricle [4].

Those patients that present following an acute LMCA occlusion are likely to have features of cardiogenic shock, carrying a grave prognosis with or without emergent revascularisation. In a case series, of acute LMCA occlusions, the presence of cardiogenic shock in AMI with LMCA occlusion occurred in 75% of cases, with all patients requiring IABP augmentation and a large proportion (42%) requiring mechanical ventilatory support prior to or during the revascularisation procedure [5].

The current literature suggests that survivors of acute LMCA occlusion often have one or more protective features which include the presence of collaterals to the LAD, a dominant RCA, absence of cardiogenic shock, and successful reperfusion [6].

The potential influence of cannabis is interesting. Animal heart models have shown that endogenous cannibinoids may mediate cardiac preconditioning, which could ameliorate the effects of ischaemia during AMI [7]. However, clinical data suggest that recent and regular use of cannabis (marijuana) is associated with increased mortality following AMI [8]. Marijuana has been shown to have important physiological effects on the cardiovascular system; such as a dose-dependent increase in resting heart of between 20–100% [9]; with an overall increase in oxygen demand and a reduction in oxygen supply seen as a result of this, and as a consequence, of an increase in carboxyhaemoglobin levels further antagonising oxygen carrying capacity. Another mechanism potentially increasing risk of MI may relate to a direct prothrombotic effect of cannabis itself, with a fivefold increase in the risk of AMI reported in the hour immediately after marijuana use [10]. 

## 3. Conclusion

LMCA occlusion is not always accompanied by classical ECG changes or haemodynamic instability, which may be moderated by the degree of collateralisation and the metabolic state. Accurate diagnosis relies on a high index of clinical suspicion and detection of subtle clues such as ST elevation in lead aVR.
